# Reviewer acknowledgement 2014

**DOI:** 10.1186/s12610-015-0020-5

**Published:** 2015-04-12

**Authors:** Roger Mieusset

**Affiliations:** CHU Hôpital Paule de Viguier, Toulouse, France

## Abstract

The Editors of *Basic and Clinical Andrology* would like to thank all of our reviewers who have contributed to the journal in Volume 24 (2014) and whose valuable support is fundamental to its success.

**Robert Aitken**

Australia

**Paolo Arosio**

Italy

**Antoine Bennet**

France

**Kim Boekelheide**

United States of America

**Guylain Boissonneault**

Canada

**Florence Boitrelle**

France

**Rafael Boscolo-Berto**

Italy

**Serge Bottari**

France

**Rui Costa**

Portugal

**Cyril Danjoux**

Canada

**Antonio Galfano**

Italy

**Mazen Ahmed Ghanem**

Egypt

**Valerie Grandjean**

France

**Jean-Francois Guerin**

France

**Safouane Hamdi**

France

**Takashi Ijiri**

Japan

**Geoffry de Iuliis**

Australia

**Clement Jimenez**

France

**Min Liu**

China

**Valerie Mitchell**

France

**Roberto Molina**

Spain

**Vassilios Papadopoulos**

Canada

**Ranjith Ramasamy**

United States of America

**Celia Ravel**

France

**Rodolfo Rey**

Argentina

**Pierre Ray**

France

**Richard Sharpe**

United Kingdom

**Rahul Sinha**

India

**Frank Tüttelmann**

Germany

**François Vialard**

France

**W. Steven Ward**

United States of America

**Chad Wayne**

United States of America

**Mahmut Yazici**

Turkey

